# The Allergic Bone Marrow? The Immuno-Capacity of the Human Bone Marrow in Context of Metal-Associated Hypersensitivity Reactions

**DOI:** 10.3389/fimmu.2019.02232

**Published:** 2019-09-18

**Authors:** Melanie J. Ort, Sven Geissler, Anastasia Rakow, Janosch Schoon

**Affiliations:** ^1^Julius Wolff Institute, Charité-Universitätsmedizin Berlin, Corporate Member of Freie Universität Berlin, Berlin Institute of Health, Humboldt-Universität zu Berlin, Berlin, Germany; ^2^Berlin Institute of Health Center for Regenerative Therapies, Charité-Universitätsmedizin Berlin, Berlin, Germany; ^3^Berlin-Brandenburg School for Regenerative Therapies, Charité-Universitätsmedizin Berlin, Corporate Member of Freie Universität Berlin, Berlin Institute of Health, Humboldt-Universität zu Berlin, Berlin, Germany; ^4^Center for Musculoskeletal Surgery, Charité-Universitätsmedizin Berlin, Corporate Member of Freie Universität Berlin, Berlin Institute of Health, Humboldt-Universität zu Berlin, Berlin, Germany

**Keywords:** delayed type hypersensitivity, metal, arthroplasty, bone marrow, immune cells, memory T cells

## Abstract

Arthroplasty ranks among the greatest achievements of surgical medicine, with total hip replacement termed “the operation of the century.” Despite its wide success, arthroplasty bears risks, such as local reactions to implant derived wear and corrosion products. Prevalence of allergies across Western society increases and along the number of reported hypersensitivity reactions to orthopedic implant materials. In this context the main focus is on delayed hypersensitivity (DTH). This mechanism is mainly attributed to T cells and an overreaction of the adaptive immune system. Arthroplasty implant materials are in direct contact with bone marrow (BM), which is discussed as a secondary lymphoid organ. However, the mechanisms of sensitization toward implant wear remain elusive. Nickel and cobalt ions can form haptens with native peptides to activate immune cell receptors and are therefore common T helper allergens in cutaneous DTH. The rising prevalence of metal-related allergy in the general population and evidence for the immune-modulating function of BM allow for the assumption hypersensitivity reactions could occur in peri-implant BM. There is evidence that pro-inflammatory factors released during DTH reactions enhance osteoclast activity and inhibit osteoblast function, an imbalance characteristic for osteolysis. Even though some mechanisms are understood, hypersensitivity has remained a diagnosis of exclusion. This review aims to summarize current views on the pathomechanism of DTH in arthroplasty with emphasis on BM and discusses recent advances and future directions for basic research and clinical diagnostics.

## Introduction

The evolution of immune systems across the plant and animal kingdom has always been an arms race between pathogen and host. Mechanisms to evade immune cells or to destroy an invader have been a fine tuned development over centuries (1). This lead to distinct lymphoid tissues in mammals, located at potential exposure sites such as skin, intestine, and pharynx. Despite these first lines of defense, other designated secondary lymphoid organs have been discovered, such as lymph nodes and spleen. In addition, the bone marrow (BM) is discussed to behave as such (2). Originally named primary lymphoid organ and producer of stem and all blood cells, the human BM is composed of monocytes, macrophages, progenitor cells, including progenitor T cells, developing B cells and mature T and B cells. In fact, the BM appears to be a major memory T cell niche maintained over years within the human body (3, 4). There is evidence that peripheral blood might not be a good representation of the individual immunological memory after all (5, 6). Memory T cells in the BM seem to have a higher proliferation rate, can be activated directly by an antigen and recruit other immune cells, thereby fostering an immune response (5). Effector memory T cells are able to migrate into inflamed tissues and further amplify this pro-inflammatory environment via their effector function, the recruitment of other immune cells, or a combination of both (7). Allergies or, more generally, hypersensitivity reactions are commonly described as an exaggeration of the immune system and categorized into four distinct types, depending on their mechanism of action and cells involved. Type I-III responses are antibody-mediated and differ in their mechanism of antigen recognition and consequent cell activation, whereas type IV hypersensitivity is T cell-mediated and an antibody-independent reaction. In order to avoid confusion with another type IV characterization, type IV hypersensitivity is termed delayed type hypersensitivity (DTH) throughout the following text. Whether allergies or at least metal induced hypersensitivity reactions occur in the peri-implant tissue is subject to global debate (8), with arguments currently lacking causal evidence. In hip and knee arthroplasty the BM, as distinct lymphoid organ, is opened and foreign bodies, the implant components, are inserted. Implant components are usually composed of ceramics, polyethylene, metals, or metal alloys and sometimes fixed with bone cement. Some of these materials, especially nickel and cobalt, which are common allergens in cutaneous hypersensitivity (9, 10) have the capacity to trigger a hypersensitivity reaction or, more precisely, an allergic reaction and consequently cause inflammation (11). Histopathological examinations of peri-implant membranes provide evidence that T cell-mediated DTH is one of the keys to clinically prevalent pathologies of implant-related inflammatory reactions (12). This review aspires to summarize current findings on the immunological capacity of the BM (13) with special regard to DTH reactions and attempts to connect the dots with metal implant material hypersensitivity and its prospects in diagnostics. The aim of this review is to raise awareness that metal hypersensitivity might occur beyond the peri-implant membrane and the individual immunological memory has to be considered.

## T Cells in Human Bone Marrow

Originally, the BM was solely described as the origin of all hematopoietic precursor cells, giving rise to lymphocytes to exit the BM and complete their maturation in the periphery. This idea has to be revised after finding a niche for memory T cells inside the BM (14–17). Mature T cells seem to migrate back to the BM to reside inside its cavities (4, 18). Progenitor T cells develop, leave the BM and migrate to the thymus, where they express their T cell receptor for the first time and get selected for the cytotoxic or the helper cell lineage by expressing either co-receptor CD8 or CD4. Once they have passed the negative and positive selection to exclude auto-reactivity in the thymic tissue; they are released into the peripheral blood. In case of infection, T cells are activated in lymphoid organs and tissues such as spleen, lymph nodes, and Peyer's patches or at the site of infection. At this specific reaction site, T helper cells interact with antigen-presenting cells (APCs) such as dendritic cells to find their cognate antigen to proliferate and migrate to the site of infection or inflammation, and stimulate other immune cells such as antibody-producing B cells. After an inflammation is terminated most T cells die and only a few home to lymphoid tissues to remain there as memory T cells. Recent findings suggest the preferred site is the BM (16). Additionally, the BM has the capacity to host all major subsets of CD4 and CD8 T cells, which include effector, central memory, effector memory, and terminally differentiated T cells (17). While effector cells are short-lived, memory T cells survive over a longer period (6). This allows for the assumption that these cells could react to antigens derived from metal implants, especially if memory had been established prior to insertion of a metallic implant. This could be the case if T cells had been primed in the periphery and remained “silent” in the BM. Yet, it needs to be established how local T cell subsets vary among humans. Naïve T cells and their travel routes through the body are poorly understood. Whereas reports on naïve T cells being primed in the BM exist (19, 20), it is unclear if naïve T cells actively migrate to the BM or just circulate between secondary lymphoid organs and within the blood (21). If the BM could act as a secondary lymphoid tissue, naïve T cells could reside there as well or at least pass through the BM and newly encounter wear particles derived from the implant. This is a concept that needs investigation, as there is currently not enough data to support or reject the idea of such a locally occurring peri-implant DTH reaction. This can only be a potential mechanism to explain why some individuals with no known sensitivities prior to surgery develop peri-implant complications (e.g., implant loosening, joint malfunction despite correct implant position) or diffuse symptoms that point towards DTH. A respective study would be of high clinical value but is not available in current literature. Roughly 2% of the total lymphocyte numbers are circulating in the periphery compared to 11% residing in the BM, of which memory T cells constitute a major part (3, 4, 16, 22). Blood values of T cells can therefore only be a rough estimate of their total numbers present in the body (5). Distinct *in vitro* cultures, such as 3D bone and BM microfluidic culture systems, could become a valuable tool to study this effect in more detail. The BM is thought to host many more memory T cells than are circulating in the peripheral blood, possibly rendering the lymphocyte transformation test (LTT), which is performed with blood T cells, a questionable predictor for metal hypersensitivity prior to implantation. It must further be noted that naïve T cells can be primed in the BM in mice (19). T cells are the main cell type to drive metal-associated delayed hypersensitivity reactions. Their presence in the BM should be kept in mind, especially when facing and treating patients with known allergies and intolerances.

## Hypersensitivity Reactions in Orthopedics

Hypersensitivity toward metals is one of the most common causes for DTH, affecting 15–20% of the Western population (23, 24) with mainly cutaneous manifestations, such as pruritus and rashes. Delayed hypersensitivity is commonly described as a local reaction in which an allergen is recognized by APCs and presented to a subset of T helper cells (Th1), which leads to a proper pro-inflammatory response. The trigger substance can react with self-proteins and form hapten-protein-complexes which bind to major histocompatibility complexes (MHC) and activate T cells like a regular foreign antigen (e.g., from bacteria). The reaction is divided in two phases: sensitization and elicitation. During sensitization the APCs home to secondary lymphoid organs and activate T cells. T cells expand and consequently produce memory T cells which trigger a stronger and more efficient response upon secondary antigen encounter. One of the most common forms of DTH is cutaneous hypersensitivity from inexpensive jewelry containing metal ions like nickel and cobalt. Nickel ions are known to induce conformational changes in the protein-MHC class II complex (25) and activate T cells, which in return release cytokines to attract macrophages to the site of allergen exposure (26). Nickel can also bind directly to the T cell receptor like a superantigen (9). This may be a reason why DTH remains systematically undetected, considering that the entire inflammatory process is locally restricted to the peri-implant region. DTH used to be a term coined by a pathomechanism which always assumes an externally inhaled, ingested, or absorbed allergen. Even though allergies to implant materials used in orthopedics are thought to occur infrequently, metal related pathologies, including peri-prosthetic osteolysis and aseptic implant loosening, rank among the most common reasons for surgical revision of arthroplasty implants (27–30). Whether implant-related hypersensitivity reactions is the underlying mechanisms has remained largely unknown. Hypersensitivity reactions induced by implant-released metals, like cobalt and nickel, have been characterized via histology, patch testing, and LTT. Issues that likely promote an underestimation of the prevalence of such allergic reactions are the lack of reliable and accurate hypersensitivity tests and a great similarity in clinical presentation with periprosthetic joint infection (PJI), another major cause of arthroplasty failure, and with a myriad of other complications in arthroplasty. Thus, typical signs and symptoms of PJI as well as of hypersensitivity include local swelling, erythema, warmth, pain, and functional deficit of the affected joint. Therefore, arthroplasty implant-related hypersensitivity has remained a diagnosis of exclusion (31). Appropriate workup must always be guided by thorough differential diagnostic thinking, directed history taking, and physical examination. Standardized histopathological examination of intraoperatively sampled “synovial-like interface membrane” (SLIM), a term summarizing synovial tissue and the periprosthetic membrane, has become a beneficial tool for determining the causes of implant failure (12). Based on histological and histochemical criteria, the expanded SLIM consensus classification differentiates the following patterns of adverse local tissue reactions to implant materials (12): Wear-induced synovitis/SLIM (type I), infection-induced synovitis/SLIM (type II), mixed synovitis/SLIM (type III), indifferent (i.e., not wear-induced, not infection-induced) synovitis/SLIM (type IV), prosthesis-associated arthrofibrosis (type V), adverse local tissue reactions to implant wear particles (type VI), and local osseous pathologies (type VII). SLIM type VI includes inflammatory reactions secondary to wear particle toxicity or host immunological hypersensitivity. This SLIM type has been found to comprise three histological patterns: (1) a predominantly macrophagic pattern with absent or minimal lymphocytic response; (2) a mixed inflammatory pattern, macrophagic and lymphocytic, with variable presence of plasma cells, eosinophils, and mast cells and (3) a granulomatous pattern, predominant, or associated with the mixed inflammatory pattern. In orthopedics, T cells infiltrating the tissue around the implant (32) are an indicator to visualize a DTH histologically. Yet, clinicians find it challenging to distinguish this from a low-grade infection. In theory the cutaneous mechanism of DTH is likely to also occur in the peri-implant tissue. The existence of DTH toward implants is being discussed controversially among researchers and physicians, but the demographics of the arthroplasty clientele are shifting toward younger, more active patients (33–35), in whom allergies have shown to be much more prevalent than in today's elderly (36, 37). So-called hypoallergenic implants, which are characterized by coating of the common metal components with inert metal-oxides or -nitrides, are treatment options for patients with known metal allergies. However, these implants have not reach overall acceptance in a clinical setup and are infrequently used. Hence reliable registry data regarding implant surveillance are pending and evidence that hypoallergenic implants actually lead to less prevalent metal-associated problems remains controversial. “Metal implant allergies” potentially promote aseptic loosening and deserve attention.

## Metal Exposure in Peri-Implant Bone Marrow

Hip replacement is the surgery of the century, allergy the topic of the decade and cobalt the allergen of the year 2016 (38). In hip and knee arthroplasty, metallic components are fixed in the acetabular bone and the femoral shaft, and the tibial plateau and shaft and the femoral shaft, respectively. Primary fixation is achieved by either a tight press-fit of the implant into the bone or by the use of bone cement. Thus, a direct contact of BM and implant components early on is inevitable and direct metal exposure of peri-implant BM cells is possible even prior to a foreign body reaction and consequent encapsulation through a collagen-rich synovial-like interface membrane (12). Moreover, the dissolution of metals within the peri-implant membrane was demonstrated by detection of these metals in the non-particulate state (39–41). The benefit of the cobalt-chromium-molybdenum (CoCrMo) alloy has been challenged due to the release of Co and Cr ions and particles and associated local and systemic adverse effects (42, 43). This alloy contains up to 1 wt% of nickel, which can also be detected systemically increased in the case of local metal release (44, 45). Notably, non-particulate metals have also been detected beyond the peri-implant membrane, in the adjacent BM of hip endoprostheses (39, 40, 46). This suggests relevant BM exposure which potentially leads to host responses in this multicellular tissue. Local immune reactions and resulting inflammatory processes have widely been described for the peri-implant membrane (47, 48). The direct contact between BM and implant as well as the permeability of the peri-implant membrane demand the reconsideration of (pre-)clinical testing of implant materials with regard to redefinition of relevant exposure sites (49).

## Adding up the Pieces

Literature on hypersensitivity reactions to orthopedic implants is largely limited to case reports and reviews well-summarizing clinical observations. In the meantime basic research is lacking or focusing on different aspects, such as identifying cell signatures and biomarkers for regeneration (50, 51). Adding up the pieces of the newly described function of the BM and T cell memory in light of implant associated aseptic complications, prevalence of allergies on the rise and changing demographics of arthroplasty patients could open up a new research field: the allergic bone marrow.

**Figure 1:**
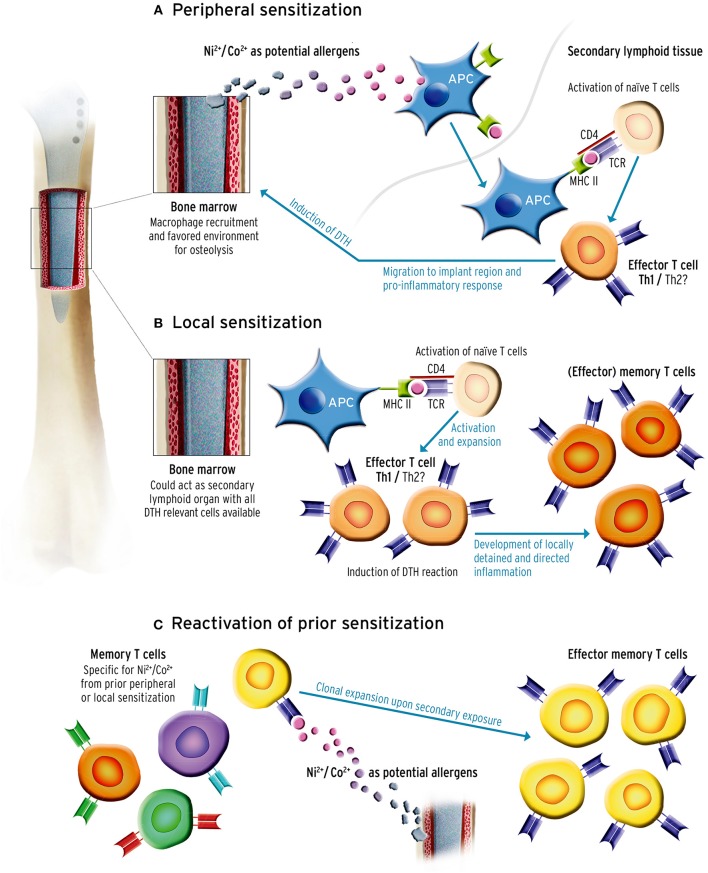
Sensitization could occur through three potential mechanisms. It is important to note that sensitization could occur prior to or after primary implantation. **(A)** Peripheral sensitization: antigen-presenting cells (APC) recognize and transport potential metal allergens derived from implant debris to the proximal lymph nodes where they present the antigen to naïve T cells. In case of recognition the T cell gets activated, clonally expands, and is recruited to the implant site in the bone marrow (BM). While effector T cells drive a pro-inflammatory response and recruit other cells, memory T cells are also formed and can be activated during secondary antigen encounter. **(B)** Murine BM can prime naïve T cells, and drive the same immune response as described in 1a only locally, without the need of leaving the BM. Since the BM could act as a secondary lymphoid organ, the naïve T cells could be primed by the present APCs, mature and clonally expand and also produce memory T cells which can be activated upon second exposure. **(C)** T cells were primed and sensitized through an exogenous antigen before primary arthroplasty. As described in **(A,B)** this produces T cell subsets including memory T cells. These could home to the BM, reside there and even remain undetected in allergy diagnostics. Once local T cells are re-exposed to implant material reassembling the antigen, the memory T cells previously sensitized with the antigen clonally expand again and drive an inflammatory response.

To begin with, the implant is brought in close contact with BM composed of immature and mature immune cells. Even months and years after surgery, lymphocytes present beyond the peri-implant membrane infiltrate the peri-implant tissues (52, 53). As mentioned earlier, T cells present in the BM may drive a hypersensitivity reaction via three imaginable mechanisms: (1) Hypersensitivities can be developed in the periphery through the same mechanism as in cutaneous sensitization: APCs transport and present potential implant-derived allergens to T cells in the proximal lymph nodes (Figure 1A). In line with this assumption are the results of post-mortem studies, showing significant amounts of metal debris in lymph nodes of patients with endoprostheses (46, 54). (2) Hypersensitivities could develop locally, where naïve T cells are primed by metal-hapten complexes and subsequently drive a directed immune response (Figure 1B). Even though there are no systematic investigations evaluating whether or not the exposure to implant-derived metal ions and particles leads to T cell priming by local APCs in the BM, recent studies indicate that the BM could act as a priming site for T cells (2, 19). Independent of peripheral or local sensitization, T cells are activated, and clonally expand and migrate to the reaction site and further drive inflammation upon chronic exposure. Another mechanism could be (3) mediated by T cells having already been primed in the periphery by an allergen, such as cobalt or nickel, prior to arthroplasty surgery. These cells home to the BM and become reactivated once implant debris occurs in the peri-implant tissue (55) (Figure 1C). Although only few studies show a correlation between higher implant revision rates and preoperative sensitization (56, 57), the prognostic value of pre-implantational cutaneous (patch test) or systemic (LTT) metal allergy testing for implant failure is still controversial (8).

**Figure 2:**
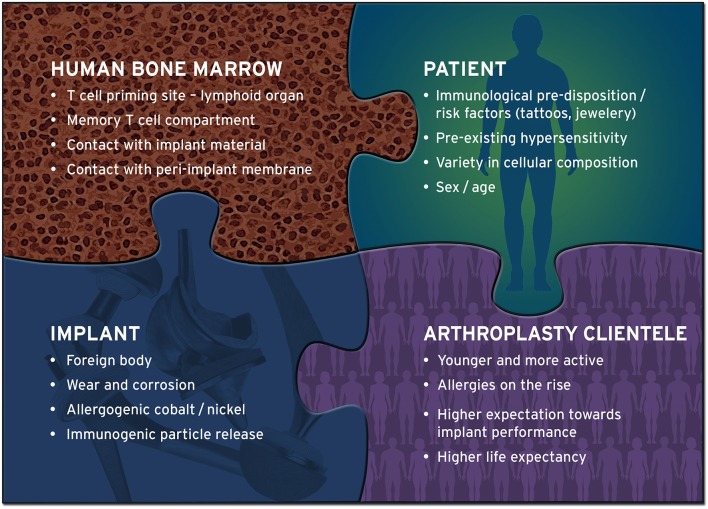
Implant-related hypersensitivity is influenced by multiple factors. Delayed type hypersensitivity (DTH) reactions to implant materials cannot be attributed to a single cause. Multiple factors influence the development and consequence of hypersensitivity, many of which are yet to be identified. Bone marrow, as a T cell harboring, possibly secondary lymphoid tissue, has the potential to develop DTH reactions when exposed to implant materials. In addition, today's arthroplasty cohort is younger, more active and thus more demanding with regard to implant longevity, and apparently more prone to allergies than the previous generation of implant carriers. These include allergies against nickel and cobalt, metals that have shown to be frequently present in the proximity of endoprostheses at the time of revision surgery.

Whether one of the proposed pathomechanisms or a combination thereof is driving the induction of a DTH reaction in the BM remains elusive and prompts for systematic investigation. Yet, the prevalence of cutaneous metal sensitivity was reported to be higher in patients with an artificial joint as compared to the general population (58). In addition, early osteolysis in a patient cohort with metal-on-metal hip replacement was positively correlated with cutaneous metal allergy (59). There is evidence that pro-inflammatory factors released during a DTH reaction, such as Il-1, IL-2, IL-6, INF-γ, and TNF along with subsequent activation of the NF-κB signaling pathway promote a distorted inflammatory environment which could enhance osteoclast activity and inhibit osteoblast function (60, 61). This imbalance is typical for osteolysis and promotes implant loosening (62). Depending on the strength of a cellular response to the antigen the respective time frame may vary from weeks to years, with only some patients showing severe allergic reactions (31). Allergies and hypersensitivity are highly individual processes and depend on multiple factors (Figure 2). Based on the evidence for the immune-modulating function of BM (6) and the higher prevalence of metal-related allergy in patients with endoprostheses, it is reasonable to presume that hypersensitivity reactions could occur in peri-implant tissues.

There seems to be an unmet clinical need for the development of a more accurate bedside diagnostic assay incorporating the potential differences between the composition of BM and peripheral blood. Such a hypersensitivity test has to be cost-effective, but above all must be rapid and easy to perform before or during surgery. Several scenarios for an effective test are conceivable: The current LTT principle for whole blood could be applied to BM cells obtained intraoperatively or, ideally, preoperatively from a patient's biopsy. Even more valuable would be a specific cellular or molecular biomarker as a determinator of possible hypersensitivity. Other tests than the LTT have been proposed, such as a cytokine ELIspot (63) which is faster compared to the LTT and easier to handle. Due to the assay time of up to 48 h such tests are currently not a realistic option for intraoperative application and would be limited to pre-operative use. It also remains to be clarified which cytokines in the BM are reliable markers for the diagnosis of DTH. The identification of such cellular or molecular biomarkers does not only require a thorough characterization of the BM and the peri-implant microenvironment, but also a mechanistic understanding of the causal relationship between the biomarker and the clinical outcome. To advance our mechanistic understanding of DTH in BM, basic research should include *ex vivo* analyses of patient samples and be combined with advanced *in vitro* models, allowing the investigation of implant-related aseptic inflammatory reactions in a human biomimetic environment.

## Concluding Remarks

Allergies are a trending topic not only in the broad media but also in the clinic and in basic research. Allergy-related health care costs and the burden on patients suffering from any kind of overreaction of the immune system are already profound. This trend will not stop at implant-related pathomechanisms. While there are many excellent reviews on the topic of metal hypersensitivity toward implant-related materials, we would like to stress the importance of new findings on human BM and its capacity to maintain mature lymphocytes and drive immune responses in regard to the potential of existing implant-related DTH. The presented mechanisms potentially underlying the development of hypersensitivity are meant as suggestions and to raise awareness regarding aseptic loosening and other complications in arthroplasty being possibly rooted in reactions induced by resident BM cells surrounding the implant. The fact that memory T cells preferentially locate in the BM niche could shed new light on an old debate and might spark new research ideas and diagnostic approaches, supporting clinicians in their decision-making.

## Author Contributions

JS developed the idea to the manuscript. MO wrote the manuscript. AR, JS, and SG edited the manuscript. All authors contributed critical feedback during the development of the manuscript and mutually discussed new findings and literature.

### Conflict of Interest Statement

The authors declare that the research was conducted in the absence of any commercial or financial relationships that could be construed as a potential conflict of interest.
